# F-MAP: A Bayesian approach to infer the gene regulatory network using external hints

**DOI:** 10.1371/journal.pone.0184795

**Published:** 2017-09-22

**Authors:** Maryam Shahdoust, Hamid Pezeshk, Hossein Mahjub, Mehdi Sadeghi

**Affiliations:** 1 Department of Biostatistics, School of Public Health, Hamadan University of Medical Sciences, Hamadan, Iran; 2 School of Mathematics, Statistics and Computer Science, College of Science, University of Tehran, Tehran, Iran; 3 National Institute of Genetic Engineering and Biotechnology, Tehran, Iran; Universitatsmedizin Greifswald, GERMANY

## Abstract

The Common topological features of related species gene regulatory networks suggest reconstruction of the network of one species by using the further information from gene expressions profile of related species. We present an algorithm to reconstruct the gene regulatory network named; F-MAP, which applies the knowledge about gene interactions from related species. Our algorithm sets a Bayesian framework to estimate the precision matrix of one species microarray gene expressions dataset to infer the Gaussian Graphical model of the network. The conjugate Wishart prior is used and the information from related species is applied to estimate the hyperparameters of the prior distribution by using the factor analysis. Applying the proposed algorithm on six related species of drosophila shows that the precision of reconstructed networks is improved considerably compared to the precision of networks constructed by other Bayesian approaches.

## Introduction

Constructing gene regulatory networks (GRNs) using microarray gene expressions data is one of the most challenging issues in bioinformatics. The noisy nature and high-dimensionality of microarray data make it difficult to find appropriate measures for characterizing gene relationships. There are various algorithm introduced for constructing gene networks. Most of them infer edges in the network by using the marginal or partial correlations between pair of genes [1–5]. The empirical sample covariance or correlation matrix is a standard tool for estimation of gene associations. However, these estimations often have poor behaviors in high-dimensional settings such as microarray datasets where the number of observations is much smaller than the number of genes [3].

It is assumed that integrating a priori information such as pathway information in a gene expressions analysis would improve the power of the method to reconstruct gene networks [6–10]. For instance, Prior Lasso method (Plasso) reconstructs the gene network using the available biological knowledge about gene interactions [6]. They use the information from Pathway Common (PC) and the Kyoto Encyclopedia of Genes and Genomes (KEGG) as prior information for the network reconstruction. Some other studies consider the joint inference of GRNs from different species gene expression dataset [11–15]. The main idea of these studies is that the GRNs of related species share common topological features with respect to a shared ancestry. Consequently, it is assumed that exploiting this common information may result in more accurate inferred network and extract the true relationships among genes regarded to determination of the conserved gene relationships among species. Although the final networks in these studies are more accurate comparing to single-species networks, but the inferred network represents just an average network rather than a species—specific one.

In this study, we aim to reconstruct a gene regulatory network by the use of the information of gene expressions profile from related species by applying Bayesian inference of precision matrix. Fitting a Gaussian Graphical Model (GGM) is a common approach to infer a gene network [16]. In the context of Gaussian graphical models (GGM), conditional relationships between a pair of genes could be inferred from partial correlations. Assuming a multivariate normal distribution for gene expression vectors, the precision matrix (the inverse of the covariance matrix of genes) could be considered as a way of estimating partial correlations. We set a conjugate Wishart prior for the precision matrix. The external information is applied in estimation of hyperparameters in prior distribution by applying a factor analysis (FA) on the covariance matrix of the related species. Then, the part of the simplified structure of the covariance matrix obtained by loading factors is used as the estimated for the hyperparameters of prior distribution. Our proposed approach (F-MAP) is then applied to the gene expressions prepared from several time points during early embryonic development in six Drosophila species. The gene networks for all six species are estimated five times. In each time the information from one of the other species is considered as external information or knowledge. The results demonstrate the effectiveness of F-MAP to exploit external hints of other species gene expressions and the improvement in the precision of the reconstructed network considerably.

## Materials and methods

### Gaussian graphical model (GGM)

Graphical models are statistical models for which a graph represents the conditional dependence structure of variables [17]. Assuming a multivariate normal distribution for a set of variables, Gaussian Graphical models (GGMs) are popular class of graphical models for modeling the conditional dependence relationships among variables through their joint distribution [18]. The precision matrix (*Θ*) could be considered as a way of representation of Gaussian Graphical Models (GGM). In this sense, each element of precision matrix (*θ*_*ij*_) demonstrates the partial correlation (*ρ*_*ij*_) between two corresponding genes.

$$\rho_{ij}=-\frac{\theta_{ij}}{\sqrt{\theta_{ii}\theta_{jj}}}$$ (1)

Therefore genes i and j would be correlated if the corresponding element in precision matrix is non-zero. In fact, non-zero elements in the precision matrix indicate the presence of direct interaction between two genes.

### Bayesian inference of precision matrix

Let Y_i_ for all i in {1, …, n} be independent and identically multivariate normally distributed observation; N (0, *Θ*^−1^) in which p×p matrix *Θ*^−1^ is an unknown covariance matrix. The likelihood function of the data *Y* = (*Y*_1_, …, *Y*_*n*_)^*T*^ is:

$$L\left(\Theta |Y\right)=\Pi_{i=1}^{n}p(Y_{i}|\Theta )\propto {\left|\Theta \right|}^{n/2}\text{exp}\left\{-\frac{n}{2}tr\left(S\Theta \right)\right\},$$ (2)

where *Θ*, the precision matrix, is positive definite. Matrix S is the sample covariance matrix and the maximum likelihood estimator (MLE) of *Θ*^−1^. The MLE is a classical estimator of *Θ*^−1^ without taking positive definite constraint into account. Additionally, the MLE is not reliable when p (the number of variables) is greater than or equal to n (number of observations)[19]. When n is smaller than p, the matrix S is no longer positive definite. Therefore, it is not possible to estimate precision matrix by *S*^−1^. A Bayesian approach is an alternative way to estimate *Θ* (and *Θ*^−1^).

#### Wishart prior distribution

By the assumption of *Y* ~ *N*(0, *Θ*^−1^) where Y is an n×p matrix, Wishart distribution is a commonly used class of distributions for *Θ* [20, 21]. Therefore, by the relationship between Wishart and inverse Wishart distribution we assume that the prior distribution on *Θ*^−1^ is the inverse Wishart distribution [22]. The prior distribution of Wishart W (ʋ, G) is:

$$P\left(\Theta \right)=\frac{1}{2^{\upsilon p/2}{\left|G\right|}^{\upsilon /2}\Gamma_{p}\left(\frac{\upsilon }{2}\right)}{\left|\Theta \right|}^{\frac{\upsilon -p-1}{2}}exp\left\{-1/2tr\left(G^{-1}\Theta \right)\right\},$$ (3)

where the scale matrix G is a p×p positive definite matrix. The parameter Г_*p*_(.), is a multivariate gamma function and the parameter ʋ is the degree of freedom which should be greater than p-1.

The parameter G can be represented as G = (ʋΩ)^-1^ in which Ω is a p×p matrix [22] and thus:

$$E[\Theta |\Omega ,\upsilon ]=\Omega^{-1}.$$ (4)

Therefore, the expectation of the covariance matrix is:

$$E[\Theta^{-1}|\Omega ,\upsilon ]=\frac{1}{\upsilon -p-1}\Omega$$ (5)

Hence, the prespecified structural form for Ω represents *structural information* about the prior mean of *Θ* and *Θ*^−1^.

The Wishart distribution is the conjugate prior for the population precision matrix of multivariate normal distribution. Thus, the posterior distribution of Θ follows Wishart distribution; W (ʋ*, (ʋ* Ω*)^*-1*^*)* in which:

$$\upsilon^{*}=\upsilon +n,\;\Omega^{*}=\;\left(\frac{n}{n+\upsilon }\right)S+\left(\frac{\upsilon }{n+\upsilon }\right)\Omega .$$ (6)

The mode of the posterior distribution (MAP) can be considered as an estimator for Θ:

$$argmax_{\Theta }p\left(\Theta |\text{Y}\right)=\left(\upsilon^{*}-p-1\right){\left(\upsilon^{*}\Omega^{*}\right)}^{-1}.$$ (7)

According to Ω*, the prior degree of freedom (ʋ) somehow represents the strength of belief about prior hyperparameters. It can be set empirically as any non-negative real number which is greater than (p-1) [20, 22–25].

The prior Wishart distribution is characterized by the hyperparameters Ω and ʋ. Estimation of these two parameters would determine the posterior distribution. In the case of existing scientific information, the hyperparameters can be specified by the investigators. Unfortunately, this prior information is rarely available. Therefore, the hyperparameters can be estimated using empirical Bayes estimation [22, 26]. In this procedure the scale matrix can be set as various forms such as an intraclass correlation or factor analysis form[22] or even it is possible to set the scale matrix as sample covariance matrix. The variability may be underestimated since the data is used twice [27]. An alternative approach is to apply a hierarchical modeling [27]. A hierarchical model is built by assigning a prior distribution on the hyperparameters. The choice of hyperprior is important. Bouriga et al. introduce hierarchical inverse Wishart prior for the estimation of covariance matrix which use the shrinkage toward diagonality [27]. In general, the hierarchical approach applies MCMC the sampling for the estimation.

In this paper, we apply the external information to estimate the hyperparameters. We estimate the hyperparameters based on the information obtained from the factor analysis on other related dataset. Actually, we apply factor analysis on other related species gene expressions data and extract the prior information to estimate Ω.

### Factor analysis (FA)

In factor analysis [28, 29] the random observed vector y of p dimension (*y*_1_, …, *y*_*p*_)^*T*^ is represented as linear combinations of a few latent variables *f*_*1*_, *f*_*2*_, *…*, *f*_*m*_ (*m*<*p*) which are called factors. For example for the *j*^*th*^ *(j = 1*, …, *n)* observation, the factor model for the *i*^*th*^ variable is:

$$y_{ij}=\mu_{i}+\lambda_{i}{}^{T}f_{j}+\varepsilon_{ij},\;i=1,\ldots ,p,$$ (8)

where *f*_*j*_ = (*f*_*j*1_, …, *f*_*jm*_)^*T*^ is a vector of factors. The coefficient vector *λ*_*i*_ = (*λ*_*i*1_, …, *λ*_*im*_)^*T*^ is called loading factor vector and its components indicate the importance of the corresponding factors. *μ*_*i*_ and *ε*_*ij*_ are the mean and the error term, respectively. This model can be written in matrix notation as:

$$y_{j}=\;\mu +\Lambda \;f_{j}+\;\varepsilon_{j},$$ (9)

The vector *y*_*j*_ is observable and none of the variables in the right-hand side of (9) are observable. It is assumed that error terms are independently distributed as normal distribution with zero mean and the variance-covariance matrix *Ψ* which is diagonal. Also, it is assumed that error terms are independent from factors. Factors can be considered as random variables or fixed quantities that vary from one individual to another. For random factors, it is assumed that *E*[*ff*^*T*^] = *Φ*. If factors are not random *Φ* is defined as: $\Phi =\;\frac{1}{n}\sum_{j=1}^{n}(f_{j}f_{j}{}^{T})$.

Taking the factor model (9) into account the covariance matrix of variables is decomposed as:

$$\sum =\Lambda \Phi \Lambda^{T}+\Psi .$$ (10)

There are different methods to estimate parameters of a factor model such as principle component methods (PCA) and maximum likelihood estimation (MLE) [29, 30]. In this work, we use the MLE approach introduced by Bai et al. [31]. Their approach considers the maximum likelihood estimation for high dimensional data where the number of variables is equal with or greater than the number of observations. They show that the MLE is able to provide more efficient estimation under large p compared to PCA which is the most frequently used approach to estimate the parameters of the factor model [32–34]. The PCA is easy to compute and provides consistent estimators for the factors and their loading coefficients when the number of variables and observations are both large [31]. Homoscedasticity of the error terms is an implicit assumption of the PCA approach. Unlike PCA, the applied MLE in our work allows the hetroskedasticities which are estimated by other parameters simultaneously.

The applied MLE uses the quasi-likelihood function since it is assumed that {*f*_*j*_} is a sequence of fixed constants. However, the analysis holds if factors assumed to be random variables. The number of factors should be prespecified. There are various methods to find the appropriate number of factors [35]. We estimate the number of factors by Bi-cross-validation for factor analysis introduced by Wang *et al*. [35]. The introduced method is based on Bi-cross-validation, using randomly held-out sub-matrices of the data to choose the optimal number of factors. The Bi-cross-validation is done by applying *esaBcv* package from software R.

The objective quasi likelihood function is:

$$ln\;L=\;-\frac{1}{2p}ln\left|\sum \right|-\;\frac{1}{2p}tr\left(S\sum^{-1}\right),$$ (11)

where, S is the sample covariance matrix. Let ∑ be as shown in (10) where *Փ* is $\frac{1}{n}\overset{}{\underset{}{\sum_{j=1}^{n}}}(f_{j}f_{j}{}^{T})$, based on the factor model (9), the corresponding quasi likelihood function is:

$$ln\;L=\;-\frac{1}{2p}ln\left|\Psi \right|-\;\frac{1}{2pn}\sum_{j=1}^{n}{\left(y_{j}-\mu -\Lambda f_{j}\right)}^{T}\Psi^{-1}\left(y_{j}-\mu -\Lambda f_{j}\right).$$ (12)

To estimate the parameters, we take the following assumptions:

**Assumption 1**. The errors are independent and identically distributed as normal distribution. *E*[*e*_*j*_] = 0 and *E*[*e*_*j*_*e*_*j*_^*T*^] = *Ψ* which is a diagonal matrix; *Ψ* = *diag*(*ψ*^2^_1_, …, *ψ*^2^_*p*_); *E*[*e*^4^_*ij*_] ≤ *C*^4^ for all i and j, for *C* ≤ ∞.
**Assumption 2**. There exists a positive large enough constant C such that:
  - ∥*λ*_*i*_∥ ≤ *C* for all i. (∥.∥ is a Frobenius norm)
  - $C^{-2}\leq \psi_{i}{}^{2}\leq C^{2}$
  - The limits ${\text{lim}}_{p\to \infty }p^{-1}\Lambda^{T}\Psi^{-1}\Lambda$ and ${\text{lim}}_{p\to \infty }\frac{1}{p}\sum_{i=1}^{p}\psi_{i}{}^{-4}(\lambda_{i}⊗\lambda_{i})\left(\lambda_{i}{}^{T}⊗\;\lambda_{i}{}^{T}\right)$ exist and ending up with positive definite matrices.
**Assumption 3**. The diagonal elements of *Ψ* are estimated in the set [*C*^−2^, *C*^2^] and Փ is restricted to be a semi-positive definite matrix with elements bounded in the interval [−*C*, *C*]. C is a large constant.

To make the factor model identifiable, Bai et al. study the ML estimation under five different identification conditions. These conditions are explained in their paper with details [31]. In this work, we only need to estimate the loadings factor matrix (*∧*) to estimate the hyperparameter Ω of the prior Wishart distribution. Therefore, we impose the identification condition which restricts Փ to an identity matrix and the matrix (*p*^−1^*∧*^*T*^*Ψ*^−1^*∧*) as diagonal one with distinct elements. The MLE is implemented via the expectation maximization algorithm (EM).

We fitt the FA model on the external data based on ML estimation using *cate* package from software R. After obtaining the loading factors, the hyperparameter Ω is estimated by:

$$\Omega ={\Lambda \Lambda }^{T}.$$ (13)

### F-MAP algorithm

**Fig 1:**
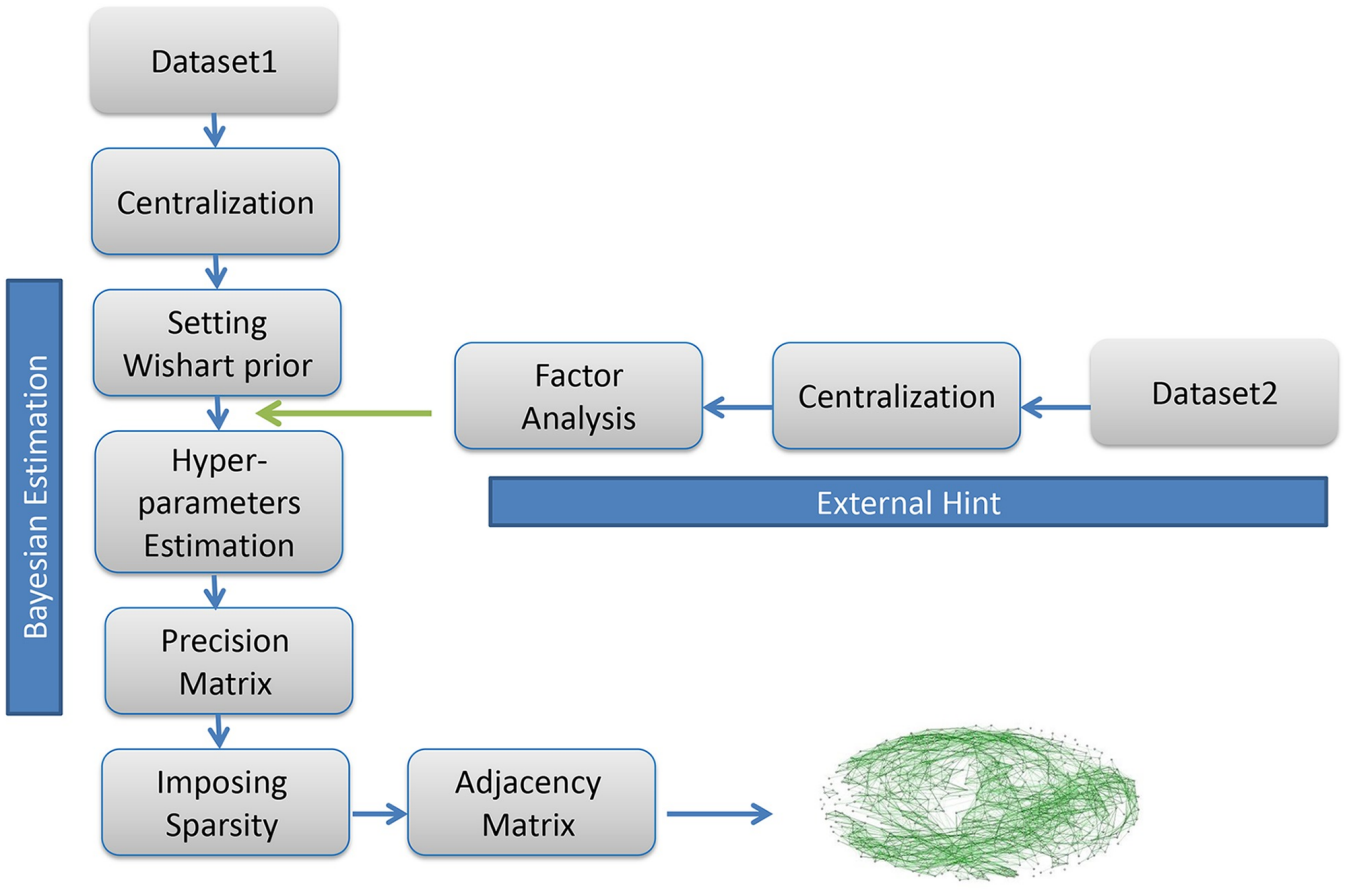
Overview of F-MAP algorithm.

Our proposed algorithm is a combination of Bayesian estimation and factor analysis which are explained above. The steps of the algorithm (Fig 1) are presented as follows:

**Step 1**. **Data pre-processing**
  There are two microarray gene expressions datasets in the algorithm. The first one is the dataset from the species which inferring its network. The other one is a dataset considered as external knowledge. The gene vectors in both datasets are centralized to have zero means.
  **Step 2**. **Bayesian inference of precision matrix**
  This step includes different stages:
  2.1 - Set Wishart prior for precision matrix; *W (ʋ*, *(ʋ*Ω*)*^*-1*^*)*
  2.2 - Choose a number greater than p-1 as the prior degree of freedom *ʋ*. The number of degrees of freedom is empirically determined (see for example [23, 24, 36]). In Eq (6) the parameter ʋ somehow represents the strength of hyperparameter Ω in the weighted average for estimating the parameter of posterior distribution, Ω*. The largest value put more weight on the hyperparameter Ω and also make the distribution concentrated around the Ω.
  2.3 - Fit a factor analysis on the covariance matrix of the related species data:
    - Determine the appropriate number of factors
    - Extract the matrix (*∧ ∧*^*T*^). It should be noted that this may lead to a non-positive definite matrix. Therefore, this problem would be solved by adding a positive value to the diagonal elements.
  2.4 - Set the hyperparameter Ω equal to matrix *∧ ∧*^*T*^.
  2.5 - Estimate the posterior distribution; W (ʋ*, (ʋ* Ω*)^*-1*^*)* by (6)
  2.6 - Estimate the precision matrix by MAP
**Step 3**. **Make sparse precision matrix**
  The posterior estimation of the precision matrix is the mode of posterior distribution (MAP) which does not have zero components. Therefore, to impose the sparsity to the MAP, the hard-thresholding method is applied. To find the threshold, we apply the decision-rule for sparse precision matrix by Kuismin and Sillanpää [24], and set the different percentiles of the absolute values of the estimated conditional correlations in the MAP as threshold and choose the one with smaller extended Bayesian information criterion (EBIC)[37].
  $$EBIC=\;-n\left\{log\left|\hat{\Theta }\right|-tr\left(\hat{\Theta }S\right)\right\}+df\left(\hat{\Theta }\right)log\left(n\right)+4df\left(\hat{\Theta }\right)\gamma log\left(p\right)\;,$$ (14)
  in which, $\hat{\Theta }$ is the sparse posterior estimate of Θ, S is the sample covariance matrix, *df* is the number of none-zero elements of $\hat{\Theta }$, *γ* is a user specified parameter which is set to 0.5 as a default value and *n* is the number of observations.
**Step 4**. **Make adjacency matrix**
  The adjacency matrix (AD) is constructed from the estimated sparse matrix in the following manner:
  $$AD_{ij}\;=\{\begin{array}{rr} 1, & {\hat{\theta }}_{ij}\neq 0\\ 0, & otherwise \end{array},$$
  where i,j = 1, …, p and p is the number of genes. The non-zero element indicates an edge between corresponding genes.

### Evaluation of the reconstructed network

In order to evaluate the performance of our algorithm in reconstruction of the network, accuracy measures; precision, recall, specificity, and accuracy are computed for each network by comparing to the gold standard network.

$$precision=\frac{\;TP}{\left(TP+FP\right)},\;recall=\frac{TP}{\left(TP+FN\right)},\;accuracy=\frac{TP+TN}{\left(TP+TN+FN+FP\right)}$$

$$specificity=\frac{TN}{\left(TN+FP\right)}\;\;.$$

Here TN is the number of true negative, FP is the number of false positive, FN is the number of false negative and TP is the number of true positive edges.

Also, for the comparative analysis, we consider the most popular as well as the state-of–the- art approaches for estimation of covariance and precision matrices; Ledoit and Wolf (Ledoit) [38] and Graphical Lasso (Glasso)[39, 40]. In order to evaluate the Bayesian framework of F-MAP with Wishart prior, we compare it with the approach by Kuismin and Sillanpää (Kuismin) [24]. Their approach uses the Wishart prior and proposes a decision-rule to estimate a sparse precision matrix.

## Results

**Fig 2:**
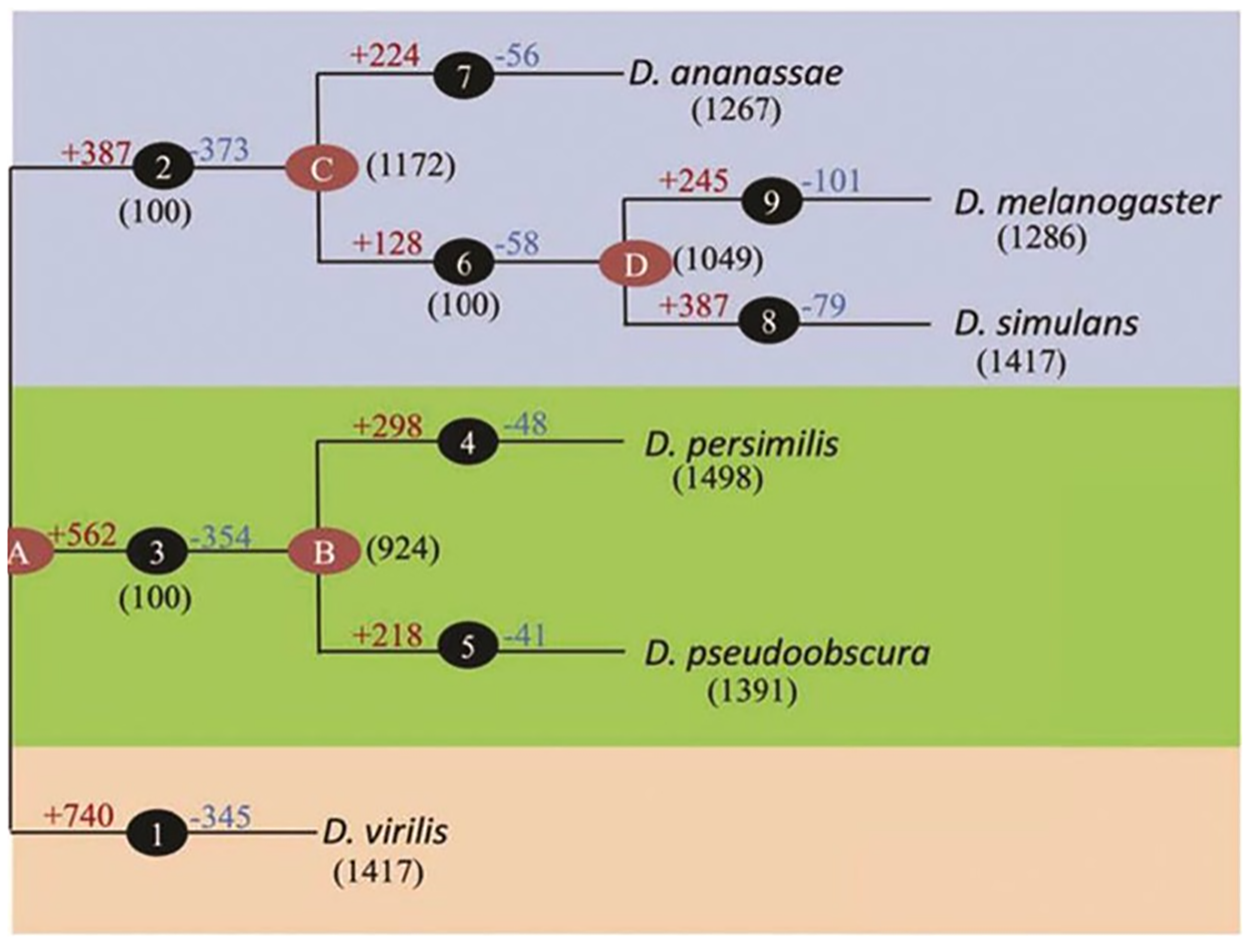
The phylogenetic tree of species. The graph is reproduced with the permission of Joshi *et al*. (2015).

We implement our approach to six datasets from six species of Drosophila fly. Dataset includes embryonic development time-course expression in six Drosophila species: *D*.*melanogaster(amel)*, *D*.*ananassa(ana)*, *D*.*persimilis(per)*, *D*.*pseudoobscura(pse)*, *D*.*simulance(sim)* and *D*.*virilis(vir)*. The phylogenetic tree between these six species is illustrated in Fig 2.

The data obtained from Kalinka *et al*. study [41] and it is accessible in *ArrayExpress* (accession code E-MTAB-404). The dataset includes the array of different developmental time points with several replicates for each species; 10 time points for *amel*, 13 time points for *vir* and 9 time points for *ana*, *per*, *pse* and *sim*. The expressions for genes are processed by averaging over absolute expression levels of different replicates and taking the log_2_ transform. We consider the expression of 2049 genes among all the dataset information. The 2049 genes are the target genes of twelve transcription factors constructing the gold standard network. Part of chip-chip data obtained from MacArthur *et al*. [42] is considered as gold standard for gene regulatory network. The chip-chip data includes 21 sequence-specific *Drosofila* transcription factors (TFs) measured in *D*.*melanogastar* embryos. For constructing a gold standard network, the information of relationships between twelve TFs which are presented on the array and their 2049 target genes are considered. The TFs and the number of their target genes are shown in Table 1. For more information of gold standard network, readers are referred to [11].

**Table 1:** Number of target genes for 12 transcription factors (TFs).

TF	zD	twi	slp1	Sna	run	prd	mad	kr	hb	dl	da	cad
Number	1166	1164	212	291	158	313	40	518	358	1503	795	273

These TF constitute the gold standard network. The gold standard network includes 6791 edges.

The specific GRN for each species is reconstructed five times by applying F-MAP. Each time, the gene expressions of one of the other species is considered as external information. The degrees of freedom for all networks are subjectively set to 2050. The degree of freedom somehow represents the strength of belief about prior hyperparameters. Since at first there is no information about which species which may improve the precision of the results, we set that as p+1. Hence, all the prior information obtained from different species will have the same weight in the posterior estimation. However, we increase it gradually and compute the accuracy measures each time. There is no considerable improvement in the accuracy measures and especially the precisions are not changed noticeably. S1 File shows the results of different degrees of freedom for each species.

To make a sparse precision matrix, the appropriate threshold for almost all the networks is set to the 95^th^ percentile of the conditional correlations in the estimated precision matrix. Thus, in order to compare the results, the threshold is chosen alike for all networks as 95^th^ percentiles of their corresponding estimated partial correlations. Therefore, the total number of edges is equal for all networks. The penalization parameters of Glasso algorithms are chosen from [0.1, 0.2, …, 0.9] to minimize the EBIC. S2 File shows EBIC for different thresholds for each method.

The diagnostic accuracy measures are computed for all methods (Table 2). All reconstructed networks almost have equal accuracy, recall and specificity. The specificity of all methods in all species are high (>90%) and the recall of all methods are 0.08 on average. The highest recall is about 15%. Comparing five reconstructed networks for a species to ones reconstructed by three other methods, shows that adding information from some species could improve the precision of reconstructed network for five species except for species *vir* (Table 2). However, as shown for the case of *vir*, the performance of our method, although lower than those for Glasso, is almost as good as the other ones. In addition, as shown by the Table 2, when using the *vir* information for network reconstruction, the precisions of the networks of other species are not affected considerably. The reason may be the different characteristics of *vir* which is solely stand in separate split of the phylogenetic tree.

**Table 2:** Measures of diagnostic accuracy of reconstructed networks for six species.

Main species	approach	Related species	Edges	True positive	Precision	Recall	Accuracy	Specificity
ana	**F-MAP**	amel	810	340	0.42	0.05	0.72	0.97
		sim	856	324	0.38	0.05	0.71	0.97
		per	1285	509	0.40	0.07	0.71	0.96
		pse*	1036	474	0.46	0.07	0.72	0.97
		vir	1721	609	0.35	0.09	0.70	0.94
	**Ledoit**		1635	590	0.36	0.08	0.70	0.94
	**Kuismin**		2230	742	0.33	0.11	0.69	0.92
	**Glasso**		480	167	0.35	0.02	0.71	0.98
amel	**F-MAP**	ana	860	393	0.45	0.06	0.72	0.97
		Sim	976	472	0.48	0.07	0.72	0.97
		per	1183	517	0.44	0.08	0.72	0.96
		pse*	1001	513	0.51	0.07	0.72	0.97
		vir	1604	612	0.38	0.09	0.71	0.94
	**Ledoit**		1647	738	0.45	0.11	0.72	0.95
	**Kuismin**		1736	758	0.44	0.11	0.71	0.94
	**Glasso**		390	207	0.53	0.03	0.72	0.99
sim	**F-MAP**	amel	802	349	0.43	0.05	0.72	0.97
		ana	819	303	0.37	0.04	0.71	0.97
		per	1246	478	0.38	0.07	0.71	0.96
		pse*	984	445	0.45	0.06	0.72	0.97
		vir	1739	633	0.36	0.09	0.70	0.94
	**Ledoit**		1550	574	0.37	0.08	0.70	0.94
	**Kuismin**		2461	968	0.39	0.14	0.70	0.9
	**Glasso**		619	274	0.44	0.04	0.72	0.98
per	**F-MAP**	ana	1595	710	0.45	0.10	0.72	0.95
		sim	1556	707	0.45	0.10	0.72	0.95
		amel*	1438	678	0.47	0.10	0.72	0.96
		pse	1761	823	0.47	0.11	0.72	0.95
		vir	2014	791	0.39	0.11	0.71	0.93
	**Ledoit**		2389	994	0.42	0.14	0.71	0.92
	**Kuismin**		1980	770	0.39	0.11	0.70	0.93
	**Glasso**		423	179	0.42	0.03	0.72	0.99
pse	**F-MAP**	ana	1318	624	0.47	0.09	0.72	0.96
		sim	1304	600	0.46	0.09	0.72	0.96
		per	1608	696	0.43	0.10	0.71	0.95
		amel*	1162	590	0.50	0.08	0.72	0.97
		vir	1959	793	0.40	0.11	0.71	0.93
	**Ledoit**		2143	932	0.43	0.13	0.71	0.93
	**Kuismin**		1859	600	0.45	0.14	0.70	0.93
	**Glasso**		432	186	0.43	0.02	0.72	0.99
Vir	**F-MAP**	ana	1951	890	0.46	0.13	0.72	0.94
		sim	2031	915	0.45	0.13	0.71	0.94
		per	2094	964	0.46	0.14	0.71	0.94
		pse*	2117	997	0.47	0.15	0.72	0.94
		amel	1881	873	0.46	0.13	0.72	0.94
	**Ledoit**		2622	1181	0.45	0.17	0.71	0.92
	**Kuismin**		2138	976	0.46	0.14	0.72	0.93
	**Glasso**		246	144	0.58	0.02	0.72	0.99

F-MAP, Ledoit and Wolf (Ledoit), Kuismin and Sillanpää(Kuismin), Graphical Lasso (Glasso).

(*): represents the species with highest impact on the network.

**Fig 3:**
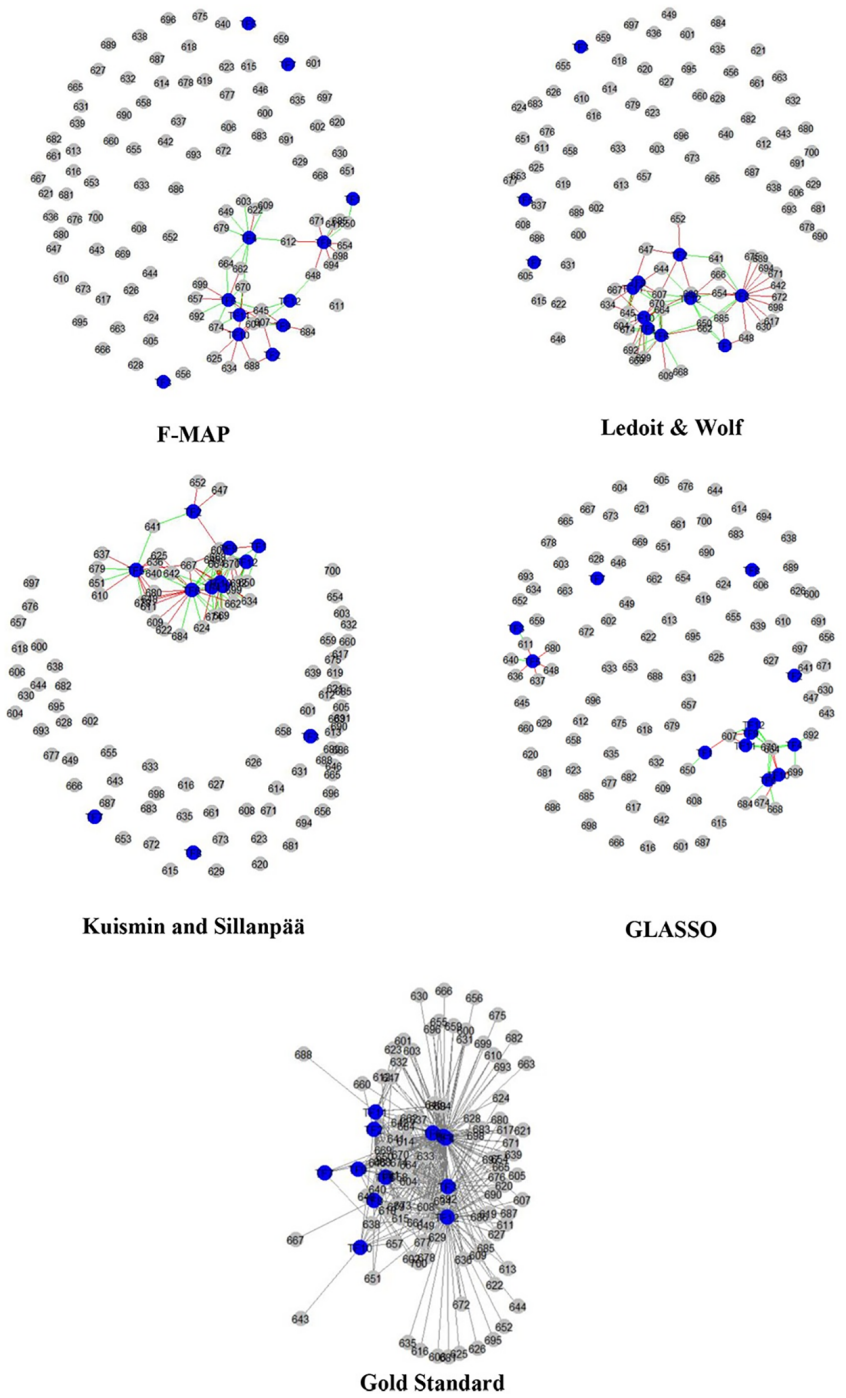
Sub-networks for *ana*. The graphs represent the interactions among 100 genes. The F-MAP network was constructed by using the information of species *pse*. The blue and grey nodes indicate the TFs and their target genes, respectively. The red and green lines indicate the false and true edges, respectively.

The highest improvement in precision is found for *ana* network reconstructed by the use of the information of *pse*. By applying the information from *pse*, the precision is found to be 46%. Since the reconstructed networks include the large number of edges, we just illustrate some parts of the final networks for *ana* (Fig 3). These graphs represent the interactions between 12 TFs and 100 genes. To choose these genes, we partitioned the gene set to 21 groups and chose the one at random. The graph for F-MAP is reconstructed by using the *pse* as external hints. All four graphs are sparser than the gold standard one. The comparison of the number of true positive (green lines) and false positives edges (red lines) between four inferred networks shows the higher precision of the F-MAP approach especially compared to Ledoit and Wolf and Kuismin and Sillanpää approaches.

**Fig 4:**
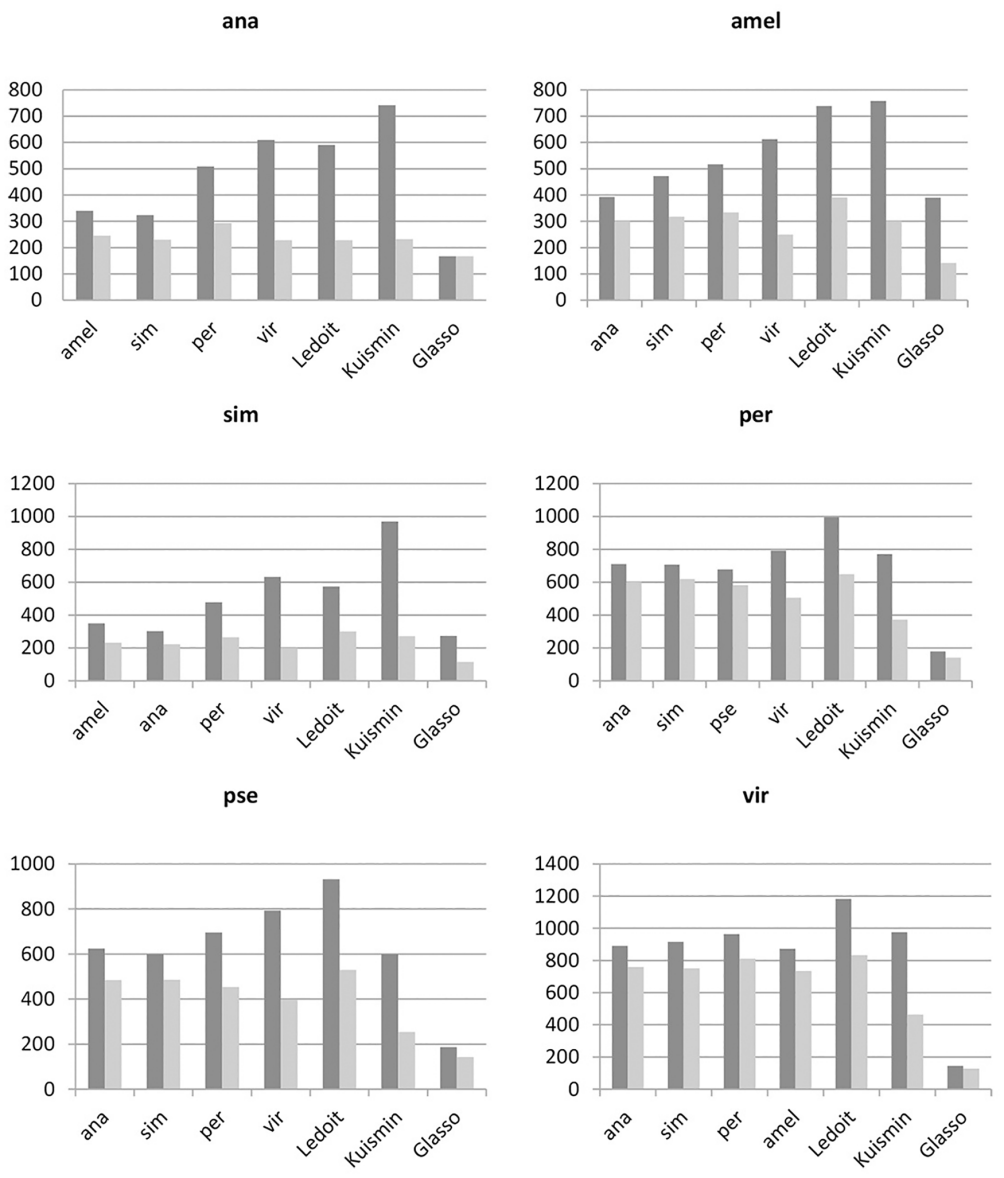
Common edges. The charts represent the number of true positive edges for each reconstructed network (black column) and the number of common edges (gray column) with the network which has the highest precision for each species in Table 2. The names of species on the horizontal axes indicate the species which its information is used as external hints for F-MAP approach. Ledoit and Kuismin represent the networks reconstructed by Ledoit and Wolf and Kuismin and Sillanpää approaches, respectively. GLASSO indicates the networks constructed by GLASSO.

Bar charts illustrated in Fig 4 represent the number of true positive edges which are common between each reconstructed networks and the one with highest precision constructed by F-MAP, for each species. Two black and grey columns of each chart represent the total number of true positive edges and the number of common edges, respectively. Comparing the height of two columns for each species shows that the networks with the highest precision include at least 40% of true positive edges of each reconstructed networks. Therefore, including the identified edges of other reconstructed network to the network with highest precision cannot improve the precision and it may just increase the false positive edges. For example, in the case of *pse*, the 81% of true positive edges of the reconstructed network by *sim* is common with those included in the network reconstructed by *amel*. Almost for all species, the least similarities often belong to the networks reconstructed by *vir* or *Kuismin* which usually have the highest number of false positive edges.

Using the information of *pse* as external hints could improve the precision of networks for all the species, especially those constructed by Ledoit and Kuismin approaches. When reconstruction of *pse* network is considered, applying the information of *amel*, *ana* and *sim* improve the precision of the network, respectively. For a more precise consideration, we simulate the *pse* dataset using 100 times sampling with replacement of the main data. Then, all the methods are implemented on all simulated data. The averages of accuracy measures for simulated data are shown in Table 3. These measures confirm that adding information of related species to *pse* improve the precision of its reconstructed networks compared to three other approaches considerably.

**Table 3:** The average of diagnostic accuracy measures of reconstructed networks for simulated data of pse.

Main species	approach	Related species	Edges (SD)	True positive (SD)	Precision (SD)	Recall (SD)	Accuracy (SD)	Specificity (SD)
**pse**	**F-MAP**	amel	990(150)	447(93)	0.45(0.03)	0.06(0.01)	0.72(0.002)	0.97(0.003)
		sim	971(189)	421(111)	0.43(0.04)	0.06(0.02)	0.72(0.004)	0.97(0.004)
		per	1560(139)	639(91)	0.41(0.03)	0.09(0.01)	0.71(0.003)	0.95(0.003)
		ana	1054(151)	469(92)	0.44(0.03)	0.07(0.01)	0.72(0.002)	0.97(0.004)
		vir	1948(102)	752(54)	0.38(0.01)	0.11(0.007)	0.70(0.002)	0.93(0.003)
	**Ledoit**		1809(371)	704(170)	0.39(0.03)	0.10(0.02)	0.71(0.005)	0.94(0.01)
	**Kuismin**		1920(320)	614(130)	0.32(0.02)	0.09(0.02)	0.70(0.003)	0.93(0.01)
	**Glasso**		373(160)	134(64)	0.35(0.05)	0.02(0.009)	0.72(0.002)	0.98(0.005)

Simulated data generated via 100 times sampling with replacement from *pse* data. SD is standard deviation of measures in 100 simulated datasets.

## Discussion

In this paper we introduce F-MAP approach; an algorithm for gene regulatory network reconstruction by using the external knowledge about gene interactions drawn from the other related species gene expressions data. Based on the results of applying F-MAP on six high-dimensional datasets, we are able to exploit external information of other species gene expressions. This in turn improves the precision of estimated network. Our approach is different from other studies which are considering the same issue [11–15], in terms of achieving a species- specific network rather than an average one.

The F-MAP is a combination of factor analysis and Bayesian frame work to estimate the precision matrix of genes in order to reconstruct the genes regulatory network. Bayesian methods to estimate the covariance matrix and consequently precision matrix such as Ledoit and Wolf method [38] focus on the computational algorithm and they are not motivated to infer gene regulatory networks. In fact, in almost all of the Bayesian approaches to estimate the covariance matrix in high-dimensional data, the condition that all the variances are the same and all the covariances are zero imposed to the estimation via determining a diagonal structure for prior hyperparameters, while the nature of a network is based on the interactions among variables. We use the factor analysis (FA) on the related species gene expressions to extract estimation of hyperparameter of the prior density function about covariance matrix. The FA gives an explanation of the interdependence of a set of genes as number of latent factors. By considering the latent factors as the elements which cause the transcription of number of genes together and also the fact that the coexpression of genes can be conserved among species, we extract the prior information of gene relationships through FA. The FA represents a simplified structure for covariance matrix, as it is shown in (10), in which covariances are modeled by loading factors. Therefore, using the factor analysis once can reduce the number of parameters.

Also, for a good factor solution the resulting partial correlations should be close to zero [43]. Therefore, another advantage of using factor analysis is that it can induce shrinkage to the posterior precision without using a restricting assumption of applying a diagonal matrix for the prior information. The F-MAP uses the hard-thresholding approach. The thresholds are chosen based on the quantiles of the estimated precision matrix. Since, genes with different factor structure have small correlations the final estimation of precision matrix will change to the sparse one.

We apply our approach on six different Drosophila fly species. To evaluate the precision of the reconstructed networks by F-MAP, three other precision matrix estimation approaches are applied; Ledoit and Wolf method and Kuismin and Sillanpää approach and Graphical lasso (Glasso). Accuracy measures of all the reconstructed networks are computed to compare the approaches. Compared to the constructed networks by these methods, The F-MAP approach can improve the *precision*. We have generated 100 simulated datasets of gene expressions of *pse* by sampling with replacement. Comparing the average of accuracy measures of the simulated data shows the considerable improvement of the precisions of F-MAP over three other methods.

Although, F-MAP has not made big increase in *recalls* but they are approximately equal to those obtained by other approaches. Actually, the true positive rates in all reconstructed networks are low perhaps reflecting that gold standard network does not contain many interactions among genes.

The other noticeable point is, although we just consider the part of the reconstructed networks based on the 12 TFS, but the differences between the numbers of edges determined by GLASSO with the size of other networks is considerably different. Especially, in the cases which GLASSO represents the highest precision, the number of edges are considerably small. These results indicate the "over-sparsity" condition imposed by GLASSO to the estimated network. However, it should be mentioned that even in the cases which GLASSO have the better performance rather than our approach, F-MAP is almost as good as GLASSO.

Although, joint graphical lasso (JGL) [44] is an extension of GLASSO for joint estimation of graphical models for the case of multiple datasets, but it is useful to determine the similarities and differences among the networks. However, the main purpose of F-MAP is to apply external information to improve the precision of the reconstructed network. That makes the F-MAP algorithm applicable in microarray studies which always deal with the high-dimensional datasets.

As the point of the time of processing, considering the high dimension of applied data, F-MAP is not time-consuming and its algorithm is processed in a few minutes. That makes the F-MAP algorithm applicable in microarray studies which always deal with the high-dimensional datasets.

As the results of applying of our approach to six different Drosophila fly show adding the information of species which is not related to the one in consideration is expected to decrease the precision. For instance, when using the *vir* information for network reconstruction, the precisions of the networks of other species are not affected considerably. However, as shown for the case of *vir*, the performance of F-MAP, although lower than those for other species, is almost as good as the other methods. Consequently, finding some criteria to choose the related species such as taking account the evolutionary distance between species could be considered. Further work is still in progress.

Also, F-MAP approach applies the information of one related species. In order to use the information of several related data, a list of related species which cause the highest improvement in precision is determined and the information about identified edges from other related species are included to its reconstructed network. This approach does not improve the precision of the network and just increase the number of false positive edges since the reconstructed network with highest precision shared at least thirty percent of true positive edges with other species. Therefore, finding a way to mix the information of several species to construct the prior information can also be of interest.

## Supporting information

S1 Fig Species *ana* subnetworks. This PDF file includes the F-MAP sub-networks for species *ana* using the information of other species.
(PDF)

S1 File Different degrees of freedom. (XLSX)

S2 File Different thresholds for making sparse matrices. (XLSX)

S3 File R codes. (ZIP)
