# Supplementary material for: F-MAP: A Bayesian approach to infer the gene regulatory network using external hints
Source: PLoS One. 2017 Sep 22;12(9):e0184795. doi: 10.1371/journal.pone.0184795 (PMC5609748; doi:10.1371/journal.pone.0184795)
Supplement: S1 Fig — This PDF file includes the F-MAP sub-networks for species ana using the information of other species. (PDF) [file pone.0184795.s001.pdf]

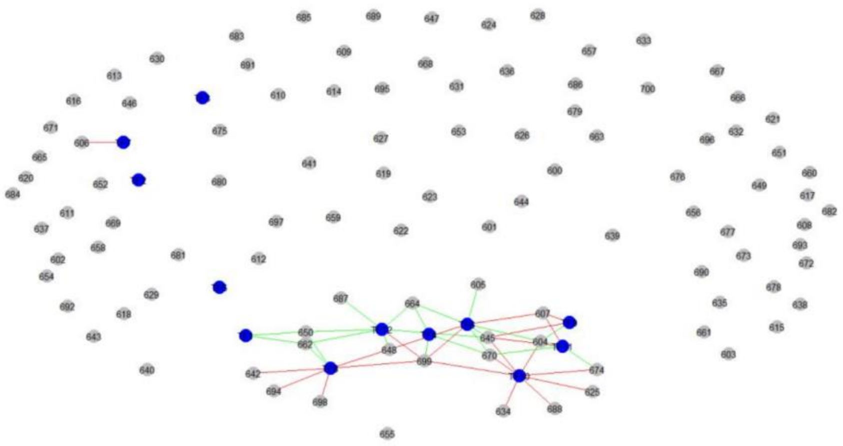

amel

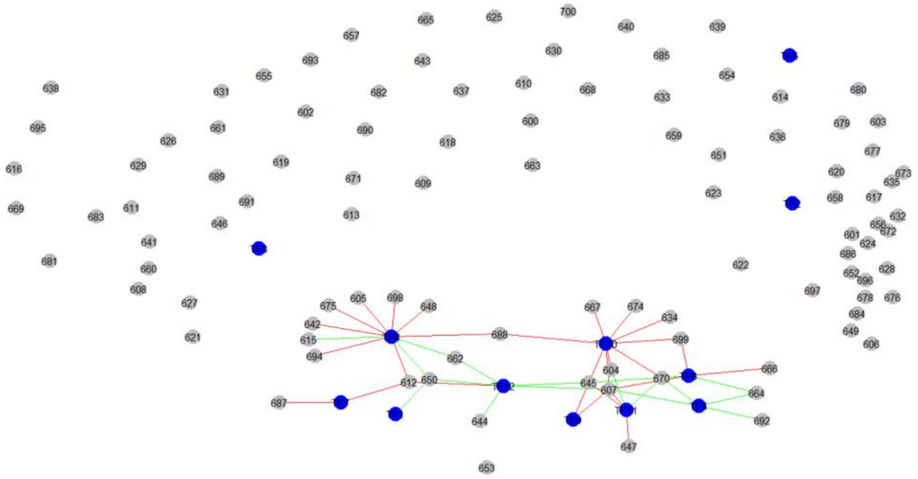

Sim

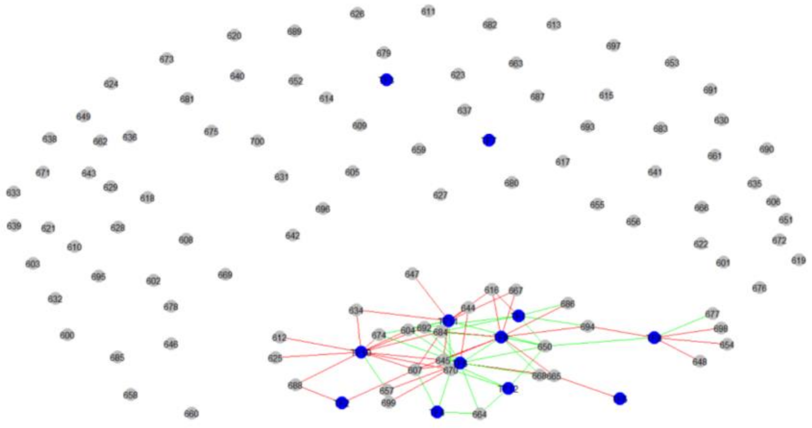

per

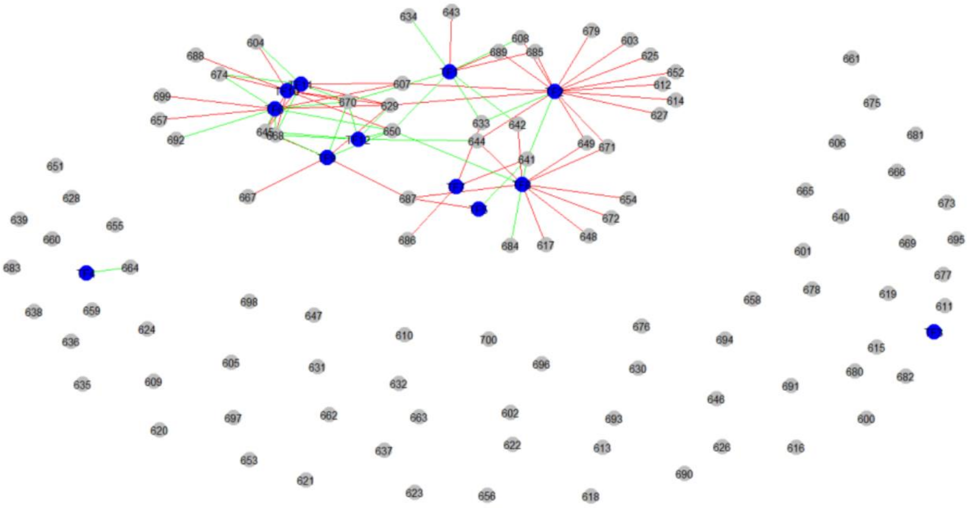

Vir

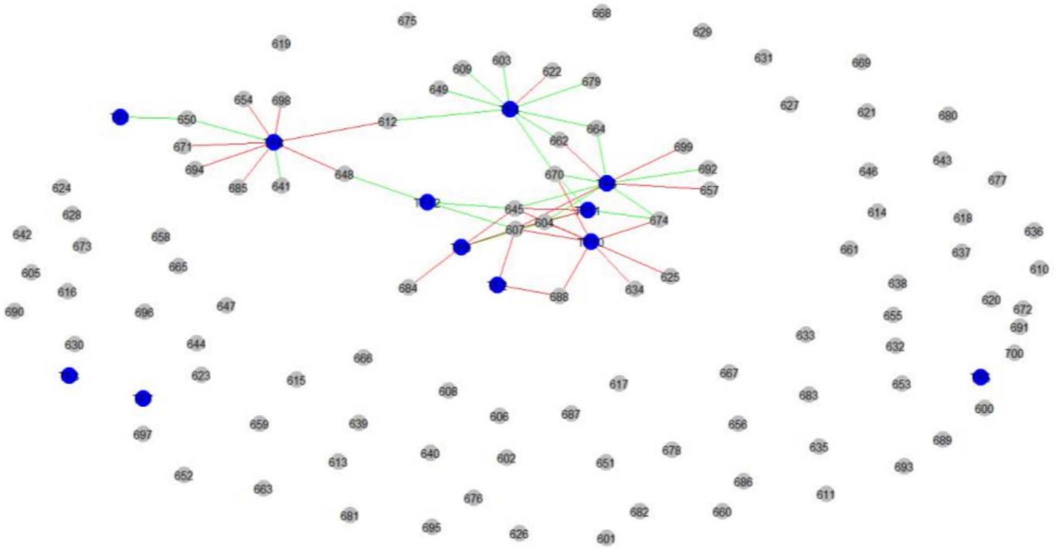

pse

Sub-networks for ana. The graphs represent the interactions among 100 genes. The F-MAP network was constructed by using the information of 5 other species. The blue and grey nodes indicate the TFs and their target genes, respectively. The red and green lines indicate the false and true edges, respectively.
