# Supplementary material for: F-MAP: A Bayesian approach to infer the gene regulatory network using external hints
Source: PLoS One. 2017 Sep 22;12(9):e0184795. doi: 10.1371/journal.pone.0184795 (PMC5609748; doi:10.1371/journal.pone.0184795)
Supplement: S3 File — (ZIP) [file pone.0184795.s004.zip › S3_File/Figure A.pdf]

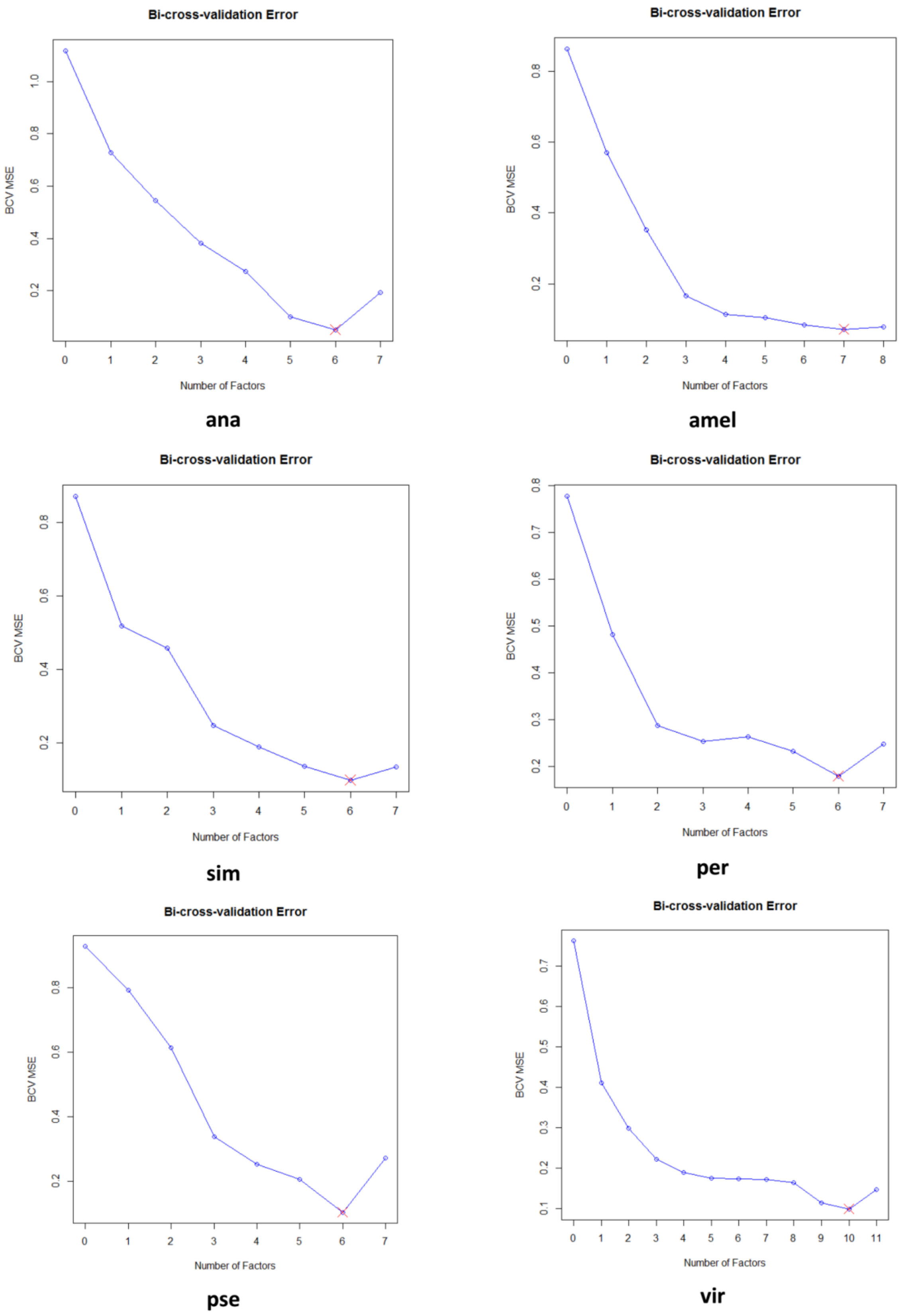

Figure A. The average Bi-Cross-Validation Error (BCV) against the number of factors.
